# Differential Proteomics Analysis Unraveled Mechanisms of *Arma chinensis* Responding to Improved Artificial Diet

**DOI:** 10.3390/insects13070605

**Published:** 2022-07-02

**Authors:** Deyu Zou, Thomas A. Coudron, Huihui Wu, Lisheng Zhang, Mengqing Wang, Weihong Xu, Jingyang Xu, Liuxiao Song, Xuezhuang Xiao

**Affiliations:** 1 Mass Production Base of Natural Enemy Insects of Tianjin Academy of Agricultural Sciences, Institute of Plant Protection, Tianjin Academy of Agricultural Sciences, Tianjin 300384, China; zdyqiuzhen@126.com (D.Z.); xwh310@163.com (W.X.); jingyang_x@163.com (J.X.); 2 Biological Control of Insects Research Laboratory, USDA-Agricultural Research Service, Columbia, MO 65203-3535, USA; coudront@missouri.edu; 3 College of Horticulture and Landscape, Tianjin Agricultural University, Tianjin 300392, China; s17698319186@126.com (L.S.); xxz24250@163.com (X.X.); 4 Key Laboratory of Integrated Pest Management in Crops, Ministry of Agriculture, Institute of Plant Protection, Chinese Academy of Agricultural Sciences, Beijing 100193, China; zhanglisheng@caas.cn (L.Z.); wangmengqing@caas.cn (M.W.)

**Keywords:** *Arma chinensis*, mass rearing, artificial diet, reformulation, nutriproteomics, biological control

## Abstract

**Simple Summary:**

*Arma chinensis* Fallou is a predaceous stink bug that can effectively control many kinds of agricultural and forest pests, such as fall armyworm, cotton bollworm and Colorado potato beetle. An insect-free artificial diet comprising chicken egg, tuna fish and raw pig liver was developed for *A. chinensis*. Several biological characteristics were diminished for *A. chinensis* reared on the artificial diet compared to the pupae of Chinese oak silk moth. Changes in the formulation of the diet were made in response to the transcriptome results and tested using biological characteristics. Several parameters were improved over 6 generations, although the improved artificial diet remained inferior to the pupae of Chinese oak silk moth regarding egg viability, fecundity, body weight, and nymphal development time. The current study reported the differential proteomic analysis revealing the mechanism of *A. chinensis* responding to the improved artificial diet. This information will be used to optimize the formulation of the artificial diet and decrease the cost of mass rearing in *A. chinensis*.

**Abstract:**

The development of artificial diets could considerably simplify and reduce the cost of mass rearing of natural enemies compared to conventional rearing methods. However, improvement of artificial diets can be tedious, convoluted and often uncertain. For accelerating diet development, a better method that can offer informative feedback to target deficiencies in diet improvement is required. Our previous research demonstrated several biological characteristics were diminished in the insect predator, *Arma chinensis* Fallou, fed on an artificial diet formulated with the aid of transcriptomic methods compared to the Chinese oak silk moth pupae. The present study reports differential proteomic analysis by iTRAQ-PRM, which unravels the molecular mechanism of *A. chinensis* responding to improvements in the artificial diet. Our study provides multivariate proteomic data and provides comprehensive sequence information in studying *A. chinensis*. Further, the physiological roles of the differentially expressed proteins and pathways enable us to explain several biological differences between natural prey-fed and improved diet-fed *A. chinensis*, and subsequent proposed reformulation optimizations to artificial diets.

## 1. Introduction

An effective artificial diet could considerably reduce the mass rearing costs of natural enemies compared to conventional rearing methods [1,2,3,4,5,6,7]. However, efforts to optimize of artificial diets can be tedious, convoluted and often result in incremental improvements. In recent years, *n*-dimensional mixture designs [8], geometric design [9], orthogonal experimental design combined with a microencapsulation technique [10] and self-selection [11,12,13,14] were also used to refine artificial diets. However, proteomic technology has not been used for reformulating artificial diet prior to this report.

The application of high-throughput sequencing of proteins combined with omics systems sciences have enabled significant advances in agricultural and nutritional research over the past decade [15]. Proteins provide nitrogen, essential amino acid for insects and key building blocks for protein homeostasis. Nutritional proteins and peptides perform key roles in various metabolisms [16]. Proteomics approaches play an increasingly important role in food authenticity [17]. Nutriproteomics could offer not only novel approaches and strategies for diet optimization but also information on diet deficiencies.

*Arma chinensis* (Fallou) (Hemiptera: Pentatomidae) is a predaceous stink bug that can effectively control many kinds of lepidopteran, hymenopteran, coleopteran and hemipteran pests [18,19,20,21,22,23,24,25]. The fall armyworm *Spodoptera frugiperda* (Smith) (Lepidoptera: Noctuidae) has caused maize yield losses as high as 50% in southern Asia and Africa [26]. Preliminary results indicate *A. chinensis* is able to suppress *S. frugiperda* populations [27,28,29].

An artificial diet comprising chicken egg, tuna fish and raw pig liver was developed for *A. chinensis* [30]. Several biological characteristics were diminished in *A. chinensis* fed on an artificial diet compared to the pupae of Chinese oak silk moth (COSM), *Antheraea pernyi* (Guérin-Méneville) (Lepidoptera: Saturniidae). Zou et al. (2013) found thousands of genes were differentially expressed between diet-fed and pupae-fed *A. chinensis* based on transcriptome information [31]. The former artificial diet was improved for continuous rearing of the predator *A. chinensis* according to transcriptome data. However, the results demonstrated further improvements may be possible. Additionally, some of the biological parameters remained consistent or improved over successive generations in insects reared on the improved artificial diet [32]. Other studies have also analyzed the relationship between gene expression patterns and diet components of predatory insects [33,34,35]. However, current genomic and transcriptomic data are insufficient to verify the exact deficiencies of the tested diets. In addition, transcript accumulation has not always been a reliable indicator of protein abundance in insects [36,37,38], because protein expression in invertebrates is strongly regulated post-transcriptionally [39]. Thus, regulations in protein level do not always depend on changes in mRNA abundance. Consequently, a more thorough picture of changes that occur during *A. chinensis* feeding on artificial diets may benefit from protein expression analyses.

Proteins represent the functional processes and biochemical machinery involved in physiological responses to dietary changes. Proteomics, an important domain of systems biology, can reveal how insects adapt to various abiotic environments, such as artificial diets. Isobaric tags for relative and absolute quantitation (iTRAQ) can help identify and quantify numerous proteins more reliably than two-dimensional electrophoresis [40]. This technique overcomes some limitations of gel-based techniques and expands the throughput of proteomic studies. In addition, the amine specific isobaric reagents permit the identification and quantitation of up to 8 different samples simultaneously [41,42]. Recently, iTRAQ has been used in insect quantitative proteomics, such as the mountain pine beetle, *Dendroctonus ponderosae* (Hopkins) (Coleoptera: Scolytidae) [43], migratory locust [44], termite [45], *Aphidius gifuensis* (Ashmead) (Hymenoptera: Braconidae) [46] and pea aphid, *Acyrthosiphon pisum* (Harris) (Hemiptera: Aphididae) [47]. Parallel reaction monitoring (PRM), which is more sensitive and specific than selected reaction monitoring (SRM), has been widely used to detect and quantify target proteins [48,49,50,51]. To date, iTRAQ discovery combined with subsequent PRM confirmation has not been used to determine key protein biomarkers in insect natural enemies. Thus, we used *A. chinensis* as the test organism to find differentially expressed proteins (DEPs) related to dietary changes by employing iTRAQ LC-MS/MS technology with PRM assays to achieve a better understanding of the molecular basis of nutrition in this study.

## 2. Materials and Methods

### 2.1. Experimental A. chinensis

The *A. chinensis* colonies used in this experiment were originally collected from Qian’an county (44°57′ N, 124°14′ E, 139 m) of Jilin province in China. The insects were reared at RH of 75 ± 5%, 27 ± 1 °C, and a 16:8 (L:D) h photoperiod. We purchased live pupae of COSM from a supermarket in Tianjin and the improved artificial diet (IAD) comprised raw pig liver, tuna fish, chicken egg and Ecuadorian shrimp [32]. Appearance and body size were used to distinguish 1st to 5th instar nymphs of F7. The F6 fertile adults, verified by hatch of their eggs, were about 15 to 20 days old. Insects were starved for 2 h, prior to analysis. Four pairs of adults, or approximately 60 nymphs from first instar to fifth instar were collected per treatment and stored in liquid nitrogen for protein extraction. Each sample had 3 biological replicates. In total, 12 pairs of adults, or approximately 180 nymphs, were used in each treatment.

### 2.2. Protein Extraction

Four samples were prepared, including COSMA (adults of *A. chinensis* fed with COSM), IADA (adults of *A. chinensis* fed with IAD), COSMN (nymphs of *A. chinensis* fed with COSM), and IADN (nymphs of *A. chinensis* fed with IAD). Each frozen sample was ground in liquid nitrogen and sonicated three times on ice using a high intensity ultrasonic processor (Scientz, Ningbo, China) in lysis buffer consisting of 2 mM EDTA, 50 mM NAM, 8 M urea, 1% protease inhibitor cocktail and 3 μM TSA. The homogenate was centrifuged at 20,000× *g* for 10 min at 4 °C. Finally, the supernatant was collected, and the protein concentration was determined with BCA (Bicinchoninic Acid) Protein Assay Reagent kit (Beyotime institute of Biotechnology, Haimen, China) according to the manufacturer’s instructions.

### 2.3. Protein Digestion and iTRAQ Labeling

For protein digestion, 10 mM DTT and 20 mM IAA were used to reduce and alkylate the protein solution at 37 °C for 1 h and at 25 °C for 45 min, respectively. For trypsin digestion, 100 mM TEAB (Triethylammonium bicarbonate) (Sigma-Aldrich, Darmstadt, Germany) was added to the protein sample until the urea concentration was less than 2 M. Trypsin was then added (*w*/*w*, protein:trypsin ratio of 50:1) and the resulting solution was held overnight. A second digestion was performed for 4 h in the ratio of 100:1 (*w*/*w*, protein:trypsin).

The tryptic peptides were desalted by Strata X C18 SPE column (Phenomenex), vacuum-dried, dissolved in 0.5 M TEAB buffer and processed according to the manufacturer’s instructions for 4-plex iTRAQ kit (AB SCIEX). Briefly, one unit of iTRAQ reagent was thawed and reconstituted in 24 μL acetonitrile (ACN). A total of 12 samples (3 biological replicates) were iTRAQ labeled. The peptide mixtures were labeled with respective isobaric tags (114 for S1 (COSMA); 115 for S2 (IADA); 116 for S3 (COSMN); 117 for S4 (IADN)), then incubated at 25 °C for 2 h and pooled, desalted and dried by vacuum centrifugation. Three biological replicates were performed.

### 2.4. HPLC Fractionation

The tryptic peptides were fractionated by high pH reverse-phase HPLC with Agilent 300 Extend C18 column (5 μm particles, 4.6 mm i.d., 250 mm length). Briefly, peptides were first separated with a gradient of 8% to 32% *v*/*v* acetonitrile (pH 9) over 60 min into 60 fractions. Then, the peptides were mixed into 18 fractions and dried by vacuum centrifuging before LC-MS/MS analysis.

### 2.5. LC-MS/MS Analysis

The tryptic peptides were dissolved in 0.1% formic acid (FA) (solvent A) and loaded onto a reversed-phase analytical column (15 cm length, 75 μm i.d, Acclaim PepMap 100, Thermo Scientific, Waltham, MA, USA). The gradient of solvent B (0.1% FA in 90% ACN) comprised an increase from 6% to 23% over 26 min, followed by 23% to 35% in the next 8 min and then up to 80% in 3 min, and held at 80% for the last 3 min, all at a constant flow rate of 400 nL/min on an EASY-nLC 1000 UPLC system.

The peptides were subjected to nanospray source followed by UPLC-MS/MS coupled online with the Thermo Scientific Q Exactive Plus platform. The electrospray voltage applied was 2.0 kV. The *m*/*z* scan range was 350 to 1800 Da for the full scan. Fixed first mass was set as 100 *m*/*z*. Intact peptides were detected in the Orbitrap at a resolution setting of 70,000. Peptides were selected for MS/MS using NCE setting of 28 and the fragments were detected in the Orbitrap at a resolution of 17,500. The data dependent acquisition (DDA) alternated between MS scans and selected for the top 20 precursor ions above a threshold intensity of 10,000 in the MS scan with 30.0 s dynamic exclusion. Automatic gain control (AGC) was used to prevent overfilling of the orbitrap with a target of 5E4 ions accumulation for generation of MS/MS spectra.

### 2.6. Database Search

MaxQuant and its integrated search engine, Andromeda (v. 1.5.2.8), were used to process the resulting MS/MS data. Tandem mass spectra were searched against *A. chinensis* transcriptome database, downloaded from the published database (NCBI SRA database under the accession numbers SRR617645 and SRR618073) and concatenated with reverse decoy database. Trypsin/P was specified as a cleavage enzyme allowing up to 2 missing cleavages and, 5 modifications per peptide. The mass tolerance was set as 5 ppm, 5 ppm and 0.02 Da for precursor ions in the First search, Main search, and for fragment ions, respectively. Carbamidomethyl on Cys was specified as fixed modification, and oxidation on Met and acetylation on protein N-terminal were specified as variable modifications. False discovery rate (FDR) thresholds were specified at 1%. Minimum peptide length was set at 7. The 4-plex iTRAQ was employed for protein quantification.

### 2.7. Bioinformatics and Statistical Analysis

The GO annotation proteome was derived from the UniProt-GOA database (http://www.ebi.ac.uk/GOA). Identified protein domain functional descriptions were annotated by InterProScan based on protein sequence alignment method and compared with the InterPro domain database. The KEGG database was employed to annotate protein pathway. WoLF PSORT was used to predict subcellular localization. A two-tailed Fisher’s exact test was performed to test the enrichment significance of DEPs against all identified proteins. Correction for multiple hypothesis testing was performed using false discovery rate control. The GO term, KEGG pathway, and protein domain categories with a corrected *p*-value < 0.05 was determined as significant. We first collated all the protein groups obtained after enrichment along with their *p* values and then filtered for categories which were at least enriched in one of the clusters with *p* value < 0.05. This filtered *p* value matrix was transformed by the function x = −log10 (*p* value). Finally, these x values were z-transformed for each category. One-way hierarchical clustering (Euclidean distance, average linkage clustering) in Genesis was then used to cluster z scores. Cluster membership was visualized by a heat map using the “heatmap.2” function from the “gplots” R-package.

### 2.8. Principal Component Analysis

The prcomp package was used to compute principal component analysis (PCA), and calculations were based on a singular value decomposition, and PCA figures were drawn by OriginPro 9.1.

### 2.9. PRM Assays

To confirm levels of DEPs determined by iTRAQ results, the expression levels of four selected proteins were quantified by a PRM-MS analysis performed at Jingjie PTM BioLab Co., Ltd. (Hangzhou, China). Signature peptides for the target proteins were defined based on the iTRAQ results, and only unique peptide sequences were determined for the PRM analysis. Details of the PRM analysis are described in Supplementary Text S1.

## 3. Results and Discussion

### 3.1. Protein Identification and Quantification

**Figure 1:**
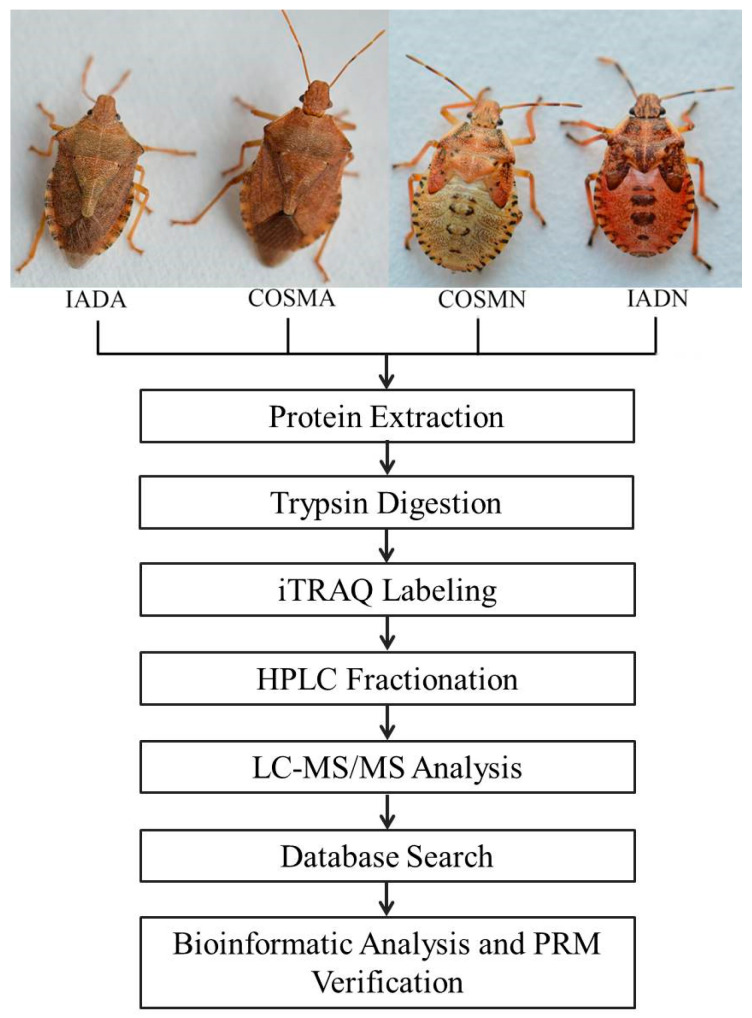
Overview of experimental procedures used in this study: (IADA) Adults of *Arma chinensis* reared on improved artificial diet; (COSMA) Adults of *Arma chinensis* reared on Chinese oak silk moth *Antheraea pernyi* pupae; (COSMN) Nymphs of *Arma chinensis* reared on Chinese oak silk moth *Antheraea pernyi* pupae; (IADN) Nymphs of *Arma chinensis* reared on improved artificial diet.

**Figure 2:**
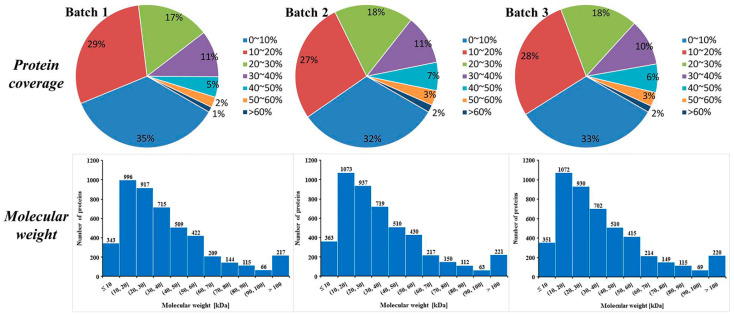
Coverage of proteins by the identified peptides and distribution of the identified proteins among the different molecular weight classes (in kD).

**Figure 3:**
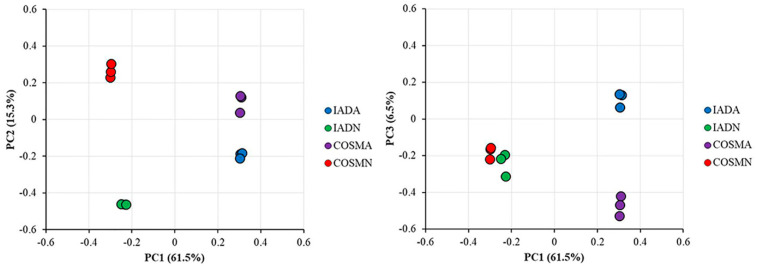
PCA of iTRAQ results in each treatment of food and developmental stage. Percentages indicate total variance explained by the first, second and third PC. The three biological replicates of a treatment are shown by dots of the same color.

A proteomic method based on iTRAQ and LC-MS/MS was applied to explore the proteomic differences between COSM-fed and IAD-fed *A. chinensis* (Figure 1). In total, three replicates of a 4-plex LC-MS/MS analysis produced 277,664, 290,026 and 285,040 spectra, corresponded to 21,770, 24,040 and 23,232 unique peptides in each replicate, respectively (See Supplementary Table S1). A total of 4653, 4795, and 4747 proteins were identified in each replicate, respectively. Most of the identified proteins (3559, 76.49%; 3669, 76.52%; and 3629, 76.45% in each of the three replicates) weighed from 10 to 60 kD (Figure 2). In addition, the identified proteins had high peptide coverage, of which 65, 68, and 67% and 36, 41, and 39% showed more than 10 and 20% sequence coverage in Batch 1, 2, and 3, respectively (Figure 2). PCA was carried out on the three replicates in each treatment to evaluate the reproducibility of the iTRAQ data (Figure 3). Results show that the iTRAQ data in three replicates in different treatments were almost unanimous, and different treatments were clearly separated, indicating that protein abundance changed with different food and different developmental stage.

### 3.2. Expression Profile of Differentially Expressed Proteins

**Figure 4:**
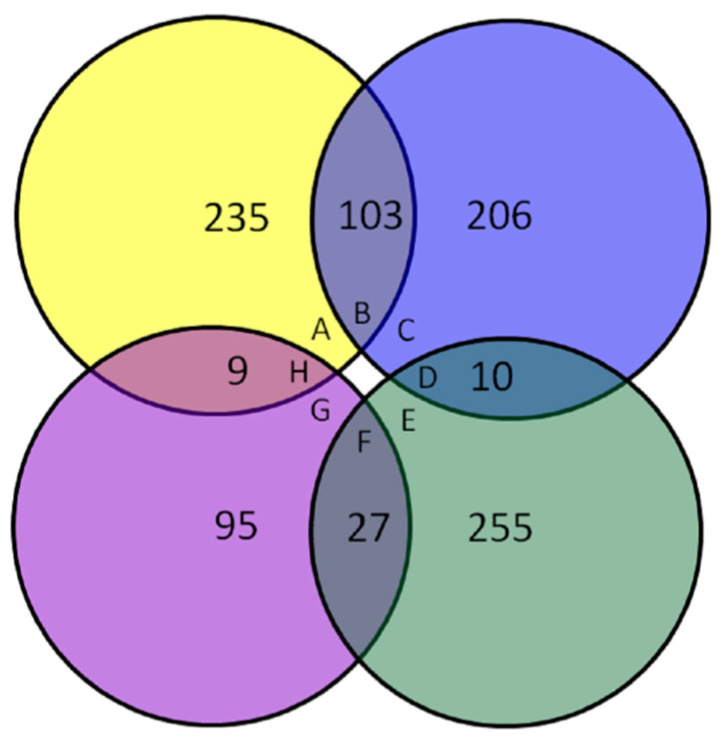
Statistical analysis of differentially expressed proteins (DEPs): A—Numbers of DEPs up-regulated only in the IADN/COSMN group; B—Numbers of DEPs up-regulated both in the IADN/COSMN group and IADA/COSMA group; C—Numbers of DEPs up-regulated only in the IADA/COSMA group; D—Numbers of DEPs down-regulated in the IADN/COSMN group but up-regulated in IADA/COSMA group; E—Numbers of DEPs down-regulated only in the IADN/COSMN group; F—Numbers of DEPs down-regulated both in the IADN/COSMN group and IADA/COSMA group; G—Numbers of DEPs down-regulated only in the IADA/COSMA group; H—Numbers of DEPs down-regulated in the IADA/COSMA group but up-regulated in IADN/COSMN group.

Proteins with corrected *p*-value of <0.05 and a fold change of >1.20 or <0.83 were considered to be significantly differentially expressed. We identified 450 DEPs, of which 319 were up-regulated and 131 were down-regulated in the IADA/COSMA group. For the 319 up-regulated DEPs, 206 DEPs were up-regulated only in the IADA/COSMA group, 103 DEPs were up-regulated both in the IADA/COSMA group and IADN/COSMN group, 10 DEPs were up-regulated in the IADA/COSMA group but down-regulated in IADN/COSMN group. For the 131 down-regulated DEPs, 95 DEPs were down-regulated only in the IADA/COSMA group, 27 DEPs were down-regulated both in the IADA/COSMA group and IADN/COSMN group, 9 DEPs were down-regulated in the IADA/COSMA group but up-regulated in IADN/COSMN group. We identified 639 DEPs, of which 347 were up-regulated and 292 were down-regulated in the IADN/COSMN group. For the 347 up-regulated DEPs, 235 DEPs were up-regulated only in the IADN/COSMN group, 103 DEPs were up-regulated both in the IADN/COSMN group and IADA/COSMA group, 9 DEPs were up-regulated in the IADN/COSMN group but down-regulated in IADA/COSMA group. For the 292 down-regulated DEPs, 255 DEPs were down-regulated only in the IADN/COSMN group, 27 DEPs were down-regulated both in the IADN/COSMN group and IADA/COSMA group, 10 DEPs were down-regulated in the IADN/COSMN group but up-regulated in IADA/COSMA group (Figure 4).

### 3.3. Functional Classification of Differentially Expressed Proteins

**Figure 5:**
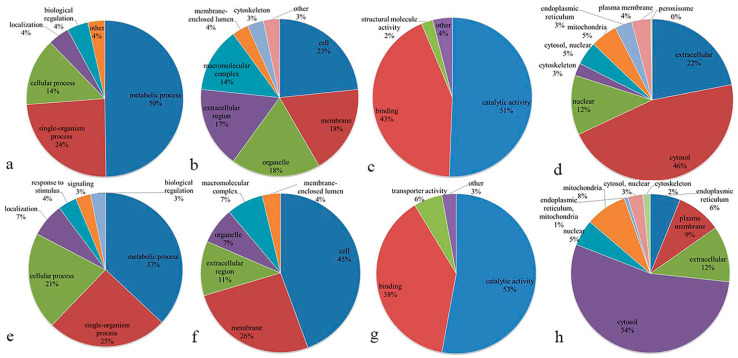
Pie charts indicating the Gene Ontology (GO) functional classification of the DEPs in IADA/COSMA group: (**a**) up-regulated DEPs categorized according to biological process; (**b**) up-regulated DEPs categorized according to cellular component; (**c**) up-regulated DEPs categorized according to molecular function; (**d**) subcellular localization of up-regulated DEPs; (**e**) down-regulated DEPs categorized according to biological process; (**f**) down-regulated DEPs categorized according to cellular component; (**g**) down-regulated DEPs categorized according to molecular function; (**h**) subcellular localization of down-regulated DEPs.

**Figure 6:**
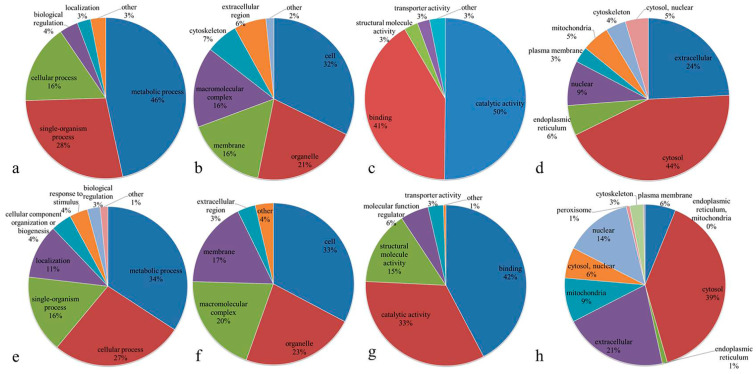
Pie charts indicating the Gene Ontology (GO) functional classification of the DEPs in IADN/COSMN group: (**a**) up-regulated DEPs classified according to biological process; (**b**) up-regulated DEPs classified according to cellular component; (**c**) up-regulated DEPs classified according to molecular function; (**d**) subcellular localization of up-regulated DEPs; (**e**) down-regulated DEPs classified according to biological process; (**f**) down-regulated DEPs classified according to cellular component; (**g**) down-regulated DEPs classified according to molecular function; (**h**) subcellular localization of down-regulated DEPs.

The Gene Ontology (GO) functional classification of the up-regulated DEPs in IADA/COSMA group based on their biological process, cellular component and molecular function (Figure 5a–c and Supplementary Table S2) indicates that the top three categories are metabolic process (50%), single-organism process (24%) and cellular process (14%) in biological process, cell (23%), membrane (18%) and organelle (18%) in cellular component, catalytic activity (51%), binding (43%) and structural molecule activity (2%) in molecular function. The GO analysis of the down-regulated DEPs in IADA/COSMA group indicates that the top three categories are metabolic process (37%), single-organism process (25%) and cellular process (21%) in biological process, cell (45%), membrane (26%) and extracellular region (11%) in cellular component, catalytic activity (53%), binding (38%) and transporter activity (6%) in molecular function (Figure 5e–g and Supplementary Table S3). The GO analysis of the up-regulated DEPs in IADN/COSMN group indicates that the top three categories are metabolic process (46%), single-organism process (28%) and cellular process (16%) in biological process, cell (32%), organelle (21%) and membrane (16%) in cellular component, catalytic activity (50%), binding (41%) and structural molecule activity (3%) in molecular function (Figure 6a–c and Supplementary Table S4). The GO analysis of the down-regulated DEPs in IADN/COSMN group indicates that the top three categories are metabolic process (34%), cellular process (27%) and single-organism process (16%) in biological process, cell (33%), organelle (23%) and macromolecular complex (20%) in cellular component, binding (42%), catalytic activity (33%) and structural molecule activity (15%) in molecular function (Figure 6e–g and Supplementary Table S5).

We also analyzed the subcellular localization of the up-regulated DEPs, and the results indicated that 147 (46%) of DEPs were located within the cytosol, 70 (22%) of DEPs were extracellular and 38 (12%) of DEPs were located within the nuclear grouping in IADA/COSMA (Figure 5d). The subcellular localization of the down-regulated DEPs in IADA/COSMA group showed that 71 (54%) of DEPs were located within the cytosol, 15 (12%) of DEPs were extracellular and 12 (9%) of DEPs were located within the plasma membrane (Figure 5h). The subcellular localization of the up-regulated DEPs showed that 151 (44%) of DEPs were located within the cytosol, 84 (24%) of DEPs were extracellular and 31 (9%) of DEPs were located within the nuclear grouping in IADN/COSMN (Figure 6d). The subcellular localization of the down-regulated DEPs showed that 115 (39%) of DEPs were located within the cytosol, 61 (21%) of DEPs were extracellular and 40 (14%) of DEPs were located within the nuclear grouping in IADN/COSMN (Figure 6h).

### 3.4. Functional Enrichment of Differentially Expressed Proteins

To analyze the enrichment tendency for DEPs’ functions, GO, KEGG pathway and protein domain enrichment analyses were performed.

#### 3.4.1. GO Enrichment

**Figure 7:**
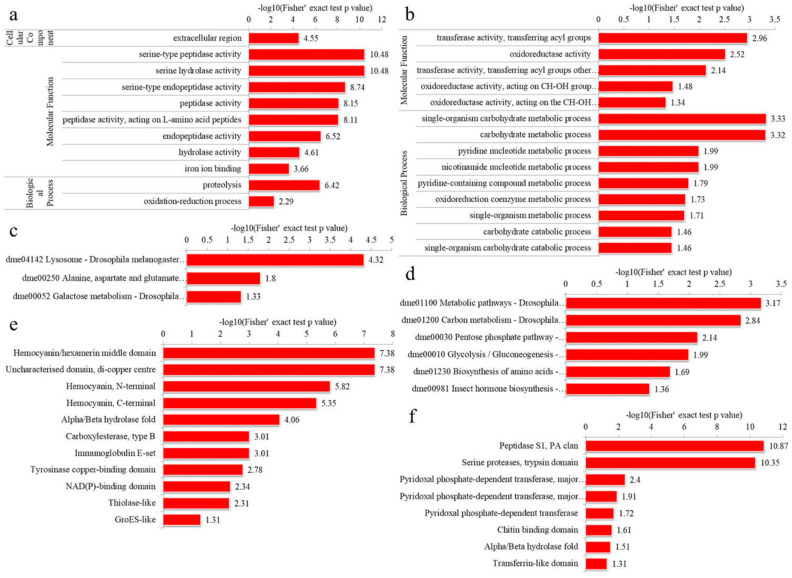
Enrichment analysis of the DEPs in IADA/COSMA group: (**a**) A GO-based enrichment analysis of the up-regulated DEPs; (**b**) A GO-based enrichment analysis of the down-regulated DEPs; (**c**) KEGG pathway enrichment analysis of the up-regulated DEPs; (**d**) KEGG pathway enrichment analysis of the down-regulated DEPs; (**e**) protein domain enrichment analysis of the down-regulated DEPs; (**f**) protein domain enrichment analysis of the up-regulated DEPs.

**Figure 8:**
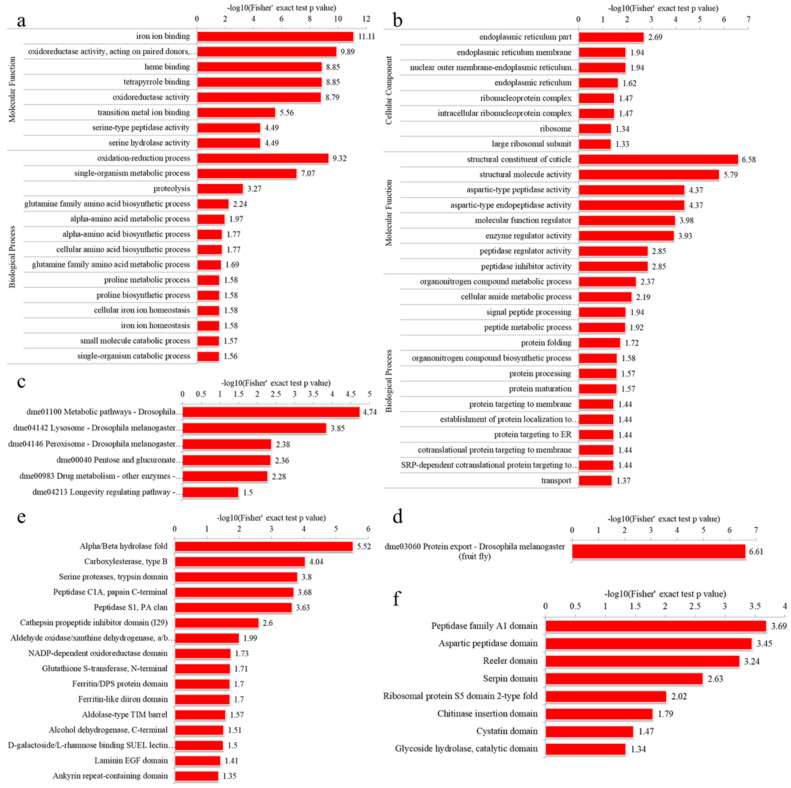
Enrichment analysis of the DEPs in IADN/COSMN group: (**a**) A GO-based enrichment analysis of the up-regulated DEPs; (**b**) A GO-based enrichment analysis of the down-regulated DEPs; (**c**) KEGG pathway enrichment analysis of the up-regulated DEPs; (**d**) KEGG pathway enrichment analysis of the down-regulated DEP; (**e**) protein domain enrichment analysis of the up-regulated DEPs; (**f**) protein domain enrichment analysis of the down-regulated DEPs.

For the up-regulated DEPs in IADA/COSMA group, most DEPs were shown to be involved in extracellular region in the cellular component category, enzymatic activity (peptidase, hydrolase and endopeptidase) and iron ion binding in molecular function category, and proteolysis and oxidation-reduction process in the biological process category (Figure 7a, Supplementary Table S6). For the down-regulated DEPs in IADA/COSMA group, most DEPs were shown to be related to transferase and oxidoreductase activity in molecular function category, carbohydrate and nucleotide metabolic process in the cellular component category (Figure 7b, Supplementary Table S7). For the up-regulated DEPs in IADN/COSMN group, most DEPs were shown to be involved in ion binding and oxidoreductase activity in molecular function category, oxidation-reduction process, single-organism metabolic process, proteolysis, and glutamine family amino acid biosynthetic process in the biological process category (Figure 8a, Supplementary Table S8). For the down-regulated DEPs in IADN/COSMN group, most DEPs were shown to be involved in endoplasmic reticulum and ribonucleoprotein complex in the cellular component category, structural constituent of cuticle, structural molecule activity, aspartic-type peptidase and endopeptidase activity in molecular function category, organonitrogen compound metabolic process, cellular amide metabolic process, signal peptide processing and peptide metabolic process in the biological process category (Figure 8b, Supplementary Table S9).

#### 3.4.2. KEGG Pathway Enrichment

The KEGG pathway enrichment analysis indicated that most up-regulated DEPs in the IADA/COSMA group were involved in lysosome, alanine, aspartate and glutamate metabolism, and galactose metabolism (Figure 7c and Supplementary Table S10). Most down-regulated DEPs in the IADA/COSMA group were related to metabolic pathways, carbon metabolism, pentose phosphate pathway, glycolysis/gluconeogenesis, biosynthesis of amino acids, and insect hormone biosynthesis (Figure 7d and Supplementary Table S11). For the up-regulated DEPs in IADN/COSMN group, most DEPs were related to metabolic pathways, lysosome, peroxisome, pentose and glucuronate interconversions, drug metabolism, and longevity regulating pathway (Figure 8c and Supplementary Table S12). The down-regulated DEPs in IADN/COSMN group were related to protein export (Figure 8d and Supplementary Table S13).

#### 3.4.3. Domain Enrichment

In the protein domain enrichment analysis, most enrichment were related to peptidase S1, PA clan, and serine proteases, trypsin domain in the up-regulated DEPs in IADA/COSMA group (Figure 7f and Supplementary Table S14). Most enrichment were related to hemocyanin/hexamerin middle domain, uncharacterized domain, di-copper centre, hemocyanin, N-terminal and C-terminal, and alpha/beta hydrolase fold in the down-regulated DEPs in IADA/COSMA group (Figure 7e and Supplementary Table S15). For the up-regulated DEPs in IADN/COSMN group, most enrichment were related to alpha/beta hydrolase fold, carboxylesterase, type B, serine proteases, trypsin domain, peptidase C1A, papain C-terminal, peptidase S1, PA clan (Figure 8e and Supplementary Table S16). Most enrichment was related to peptidase family A1 domain, aspartic peptidase domain, reeler domain, serpin domain, ribosomal protein S5 domain 2-type fold, chitinase insertion domain, cystatin domain, and glycoside hydrolase, catalytic domain in the down-regulated DEPs in IADN/COSMN group (Figure 8f and Supplementary Table S17).

### 3.5. Validation of DEPs by PRM

**Figure 9:**
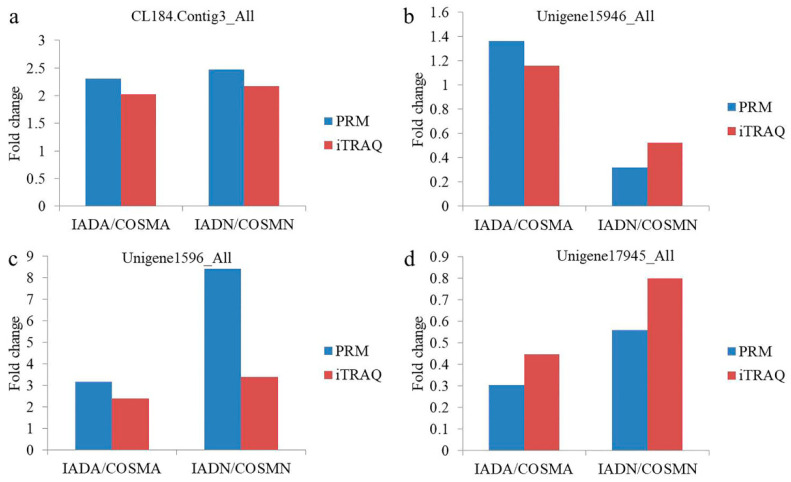
Relative expression levels of selected proteins obtained using iTRAQ and PRM in the IADA/COSMA and IADN/COSMN: (**a**) CL184.Contig3_All (minus strand secreted salivary trypsin); (**b**) Unigene15946_All (seminal fluid protein CSSFP042); (**c**) Unigene1596_All (ferritin); (**d**) Unigene17945_All (minus strand odorant-binding protein RproOBP2 precursor).

The PRM analysis is more sensitive and specific than selected reaction monitoring (SRM) and has been widely used to quantify and detect target proteins [49,52,53]. The PRM analysis succeeded in detecting 4 DEPs (CL184.Contig3_All, Unigene15946_All, Unigene1596_All, and Unigene17945_All) from iTRAQ involving 9 unique peptides (Table 1). The results indicated that the expression levels of the 4 DEPs in the different comparison programs were basically consistent with those in the iTRAQ data (Figure 9). The difference between the expression levels is likely a result of the different detection methods used [52,54]. Therefore, the PRM analysis validates that our iTRAQ data were reliable.

### 3.6. DEPs and KEGG Associated with Different Biological Parameters

Several biological parameters differed between COSM-fed and IAD-fed insects, such as lower egg viability, reduced fecundity, prolonged nymphal development time, higher cannibalism and longer lifespan [30,32]. Juvenile hormone (JH) is used by most adult insects for regulation of various aspects of reproductive maturation and behavior [55,56]. The precise nature of its role varies with the insect and its particular reproductive strategy. In our study, DEPs enriched in the pathway of insect hormone biosynthesis involved in JH were down-regulated in IAD-fed *A. chinensis*, including JH-III synthase and JH epoxide hydrolase catalyzing four chemical reactions (Supplementary Figure S1). Thus, the down-regulated JH-III synthase and JH epoxide hydrolase in this pathway could contribute to reduced fecundity.

The target-of-rapamycin (TOR) has been found to respond to the presence of amino acids and induce up-regulation of ribosome biogenesis, translation [57,58,59,60,61] and energy metabolism [62,63] required for tissue growth. mTOR is highly conserved in eukaryotes [64,65] and generally promotes cell division and cellular growth triggered by nutrient and growth factor cues [66,67,68]. The mTOR protein generally plays a role in the mTOR complex 1 (mTORC1) and mTOR complex 2 (mTORC2). mTORC1 has been found to repress autophagy, promote global mRNA translation, and modulate mitochondrial metabolism, with each of these downstream functions involved in its role in aging [68]. Increased life span in nematode *Caenorhabditis elegans* (Rhabditida: Rhabditidae) [69,70], and vinegar fly *Drosophila melanogaster* (Meigen) (Diptera: Drosophilidae) [71,72] has been found to be closely related to reduced mTORC1 signaling.

In our study, mTOR enriched in the longevity regulating pathway related to autophagy was up-regulated in IAD-fed nymphs (Supplementary Figure S2). Autophagy promotes recycling of cellular components and degradation through a lysosome-dependent regulated mechanism. In addition, it plays an important role in the homeostasis of non-starved cells. The up-regulated mTOR in IAD-fed nymphs likely repressed autophagy and recycling of cellular components, which could contribute to lower nymphal weight, shorter nymphal body length for diet-fed nymphs. In addition, catalase (CAT, CTL-1/2, CTT1) [73,74,75], superoxide dismutase, Cu-Zn family (SOD1) (Protein accession: Unigene14958_All and Unigene11805_All) [76,77], heat shock 70 kDa protein 1/2/6/8 (HSPs) (Protein accession: Unigene17363_All, IADN/COSMN ratio = 2.707, *p* = 0.031) related to longevity [78,79] were all up-regulated in IAD-fed nymphs in this study (Supplementary Figure S2), which could contribute to longer longevity for diet-fed adults. We did not find DEPs enriched in the longevity regulating pathway in IAD-fed adults. However, SOD1 (Protein accession: Unigene14958_All, IADA/COSMA ratio = 1.870, *p* = 0.024) were significantly up-regulated in IAD-fed adults, which could contribute to longer longevity for diet-fed adults.

Approximately half of all proteins expressed in eukaryotic cells are transferred into or across at least one cellular membrane to perform their functions. Protein translocation into the endoplasmic reticulum (ER) is critical to the subsequent localization of secretory and transmembrane proteins. As an essential component of the translocation machinery, the signal peptidase complex (SPC) cleaves the signal peptide sequence (SP) of secretory and membrane proteins entering the ER [80]. Failure to cleave the SP leads to the accumulation of unprocessed proteins in the ER [81]. SEC11 is the catalytic component of SPC, which catalyzes the cleavage of N-terminal signal sequences of proteins targeted to the ER. Signal peptide cleavage occurs during the translocation (co-translationally or post-translationally) through the translocon pore into the ER [82,83,84,85]. On the whole, in our study, SEC61α, SEC61β, BiP, SRP72, SRP54, SRPR, SPCS1, SPCS2 and SEC11 enriched in the pathway of protein export were significantly down-regulated in IAD-fed nymphs (Supplementary Figure S3), which could contribute to longer developmental time, shorter body length and lower body weight for diet-fed nymphs.

Vitellogenin is a female-specific egg yolk protein, with a key function linked to oogenesis [86]. Two DEPs related to vitellogenin were up-regulated in IAD-fed *A. chinensis*. However, two seminal fluid proteins (Sfps) were down-regulated in IAD-fed insects, although the expression level of minus strand seminal fluid protein CSSFP066 did not differ significantly between IAD-fed and COSM-fed *A. chinensis* (Supplementary Table S18). Sfps of male insects are transferred to females during mating and induce numerous behavioral and physiological post-mating changes in females. These changes include increasing egg production; affecting sperm storage parameters and the extent of post-copulatory sexual selection; decreasing receptivity to remating; and modulating sperm competition, feeding behaviors, and mating plug formation [87,88,89,90]. Therefore, the down-regulated Sfps in the IAD-fed insects could contribute to lower egg viability and reduced fecundity.

Four DEPs, minus strand odorant-binding protein RproOBP2 precursor, odorant-binding protein RproOBP6 precursor, odorant binding protein and odorant-binding protein 3 were down or up regulated in the diet-fed *A. chinensis*, indicating sensory ability to odors was affected (Supplementary Table S18).

Additionally, 25 and 7 DEPs related to cuticle were down and up regulated in IAD-fed nymphs and IAD-fed adults, respectively, which may have contributed to the longer developmental time, lower body weight and shorter body length for diet-fed nymphs, and to the lower body weight and shorter body length for diet-fed adults (Supplementary Table S18).

### 3.7. DEPs and KEGG Associated with Artificial Diets

One important objective of this study was to find information on diet limitations and offer a means to optimize the artificial diet. We improved artificial diet with nutriproteomics methods mainly based upon relationships between protein (enzyme) expression patterns and nutrients and KEGG pathway enrichment analyses involving nutrients. By applying basic biochemistry premises, adjusting the diet formulation by reducing the substrate, or increasing the product of an enzyme upregulated in diet-fed insects, should result in an improved formulation. In contrast, adjusting the diet formulation by increasing the substrate, or decreasing the product, of an enzyme downregulated in the diet-fed insects should result in an improved formulation. Similarly, when several enzymes within a KEGG pathway are affected in diet-fed insects, adjusting the concentration of the initial substrate of the pathway can be altered to achieve the desired adjustment in the expression of enzymes in the pathway [91].

Six DEPs enriched in the pathway of biosynthesis of amino acids related to histidine, tryptophan, tyrosine and phenylalanine were down-regulated in IAD-fed adult insects, including transaldolase, fructose-bisphosphate aldolase, class I, glyceraldehyde 3-phosphate dehydrogenase, enolase, L-serine dehydratase and L-serine/L-threonine ammonia-lyase. This indicated that more histidine, tryptophan, tyrosine and phenylalanine were needed in the IAD for adult insects. However, aspartate aminotransferase involved in glutamine were up-regulated in IAD-fed adult *A. chinensis*. Because L-glutamine was added to the diet separately, the concentration of L-glutamine in the diet could be increased independently (Supplementary Figure S4).

All five DEPs enriched in the pathway of glycolysis/gluconeogenesis involved in sucrose and starch metabolism were down-regulated in IAD-fed adult *A. chinensis*, including fructose-1,6-bisphosphatase I, fructose-bisphosphate aldolase, class I, glyceraldehyde 3-phosphate dehydrogenase, enolase and alcohol dehydrogenase 1/7 (Supplementary Figure S5). All four DEPs enriched in pentose phosphate pathway were also down-regulated in IAD-fed adult insects, including 6-phosphogluconate dehydrogenase, fructose-1,6-bisphosphatase I, transaldolase and fructose-bisphosphate aldolase, class I (Supplementary Figure S6). This indicated that more sucrose or starch was needed in the IAD for adult insects. However, all six DEPs enriched in the pathway of pentose and glucuronate interconversions related to amino sugar and nucleotide sugar metabolism were up-regulated in IAD-fed nymphs, including glucuronosyltransferase, beta-glucuronidase, UDP glucose 6-dehydrogenase, UTP-glucose-1-phosphate uridylyltransferase, aldehyde reductase and L-iditol 2-dehydrogenase (Supplementary Figure S7). These differentially expressed nutrient-controlled enzymes indicated that sucrose should be reduced in IAD for nymphs.

Several DEPs enriched in lysosome related to breakdown of protein, fat, nucleic acid and polysaccharide were up-regulated in IAD-fed adults and nymphs, respectively (Supplementary Figures S8 and S9), which could indicate that some ingredients in this artificial diet were nondigestible. Three DEPs enriched in peroxisome involved in fat degradation were up-regulated in IAD-fed nymphs (Supplementary Figure S10). Peroxisomes play important roles in the conversion of reactive oxygen species and lipid metabolism in the cytoplasm [92,93]. These three up-regulated enzymes demonstrated that saturated fatty acid or animal fat should be reduced in IAD for nymphs.

Inositol monophosphatases were up-regulated in both IAD-fed adults and nymphs, indicating inositol potentially could be increased in the diet for both nymphal and adult stages (Supplementary Table S19). Pyridoxine/pyridoxamine 5’-phosphate oxidase was down-regulated in IAD-fed nymphs, indicating pyridoxine could be increased in the diet for nymphs (Supplementary Table S19). Riboflavin kinase was up-regulated in IAD-fed adults, indicating that riboflavin could be reduced in the diet for adult stage (Supplementary Table S19). Thiamin pyrophosphokinase was up-regulated in IAD-fed nymphs, indicating thiamin could be reduced in the diet for nymphs (Supplementary Table S19).

Zinke et al. (2002) classified differentially expressed nutrient-controlled genes in larvae of vinegar flies into groups representing different physiological pathways mediating fat metabolism, such as acyl CoA thioesterhydrolase, acetyl CoA carboxylase, ATP-citrate lyase, glucose-6-phosphate dehydrogenase, triacylglycerol lipases, and Zwischenferment, as well as sugar metabolism such as glucose transporter, lipase 3, fatty acid synthase, and insulin receptor [94]. For IAD-fed insects, fatty acid synthases were found to be down-regulated in the IAD-fed adults but up-regulated in the IAD-fed nymphs (Supplementary Table S19). These differentially expressed nutrient-controlled proteins again indicated that sucrose should be reduced in the IAD for nymphs but increased in the IAD for adults.

To perform physiological activity, nutritional protein or peptide must be digestible and reach a target site in the insect body at a sufficient concentration. In our study, secreted salivary trypsin was up-regulated in IAD-fed insects which again indicated that some ingredients of IAD, especially proteins, were not easy to digest (Supplementary Table S19). Metallothionein-1F and albumin that matched the proteins from pig *Sus scrofa* (Linnaeus) (Artiodactyla: Suidae) were up-regulated in IAD-fed *A. chinensis*, which demonstrated that pig liver should be reduced in IAD for both nymphs and adults (Supplementary Table S19).

In our previous work, we sequenced 4.79 and 4.70 Gb of the transcriptome from COSM-fed and artificial diet-fed *A. chinensis* libraries, respectively, of which, nymphs and adults were collected as one sample for RNA extraction [31]. In this study, nymphs and adults were collected separately for protein extraction. The physical range of *A. chinensis* nymphs is limited. However, adults are more mobile and can acquire a wider range of prey species. In addition, *A. chinensis* adults need more nutrition in mating and reproduction. Consequently, there is likely to be a benefit in having different diet formulations for nymphs and adults. Our proteomic analysis presents DEPs with different regulated directions and/or different KEGG pathways between nymphs and adults fed on the same IAD, which indicates that nymphs and adults have different nutritional requirements. The next step is to test life history parameters in nymph and adult *A. chinensis* fed specialized artificial diets with formulations improved according to the findings we report here.

## 4. Conclusions

We present the first report of differential proteomic analysis by iTRAQ-PRM of *A. chinensis* responding to improved artificial diet and found that DEPs caused by food changes were involved in physiological differences observed in IAD-fed and COSM-fed *A. chinensis*. Several DEPs and KEGG pathways related to these different life history parameters were found. Futhermore, we found some metabolic pathways related to nutrition and differentially expressed nutrient-controlled proteins that may provide suggestions for possible formulation improvements. Findings of our proteomic analyses demonstrated direct correlations between diet formulations and biological parameters. These results indicate a nutriproteomic approach holds promise for deciphering the molecular mechanism of food changes in natural enemies and for optimizing diet formulations.

## Figures and Tables

**Table 1:** Confirmation of DEPs detected in iTRAQ analysis using PRM analysis.

Protein Accession	Protein Description	Peptide Sequence	Fold Change (IADA/COSMA) in PRM	Fold Change (IADN/COSMN) in PRM
CL184.Contig3_All	minus strand secreted salivary trypsin	EVSCPCGWTNK	2.303 (*p* = 0.0007)	2.476 (*p* = 0.0058)
Unigene15946_All	seminal fluid protein CSSFP042	ELFYISNSQR	1.363 (*p* = 0.0863)	0.32 (*p* = 0.0023)
		FQPESTSK		
		TNFAGDSAGALK		
Unigene1596_All	ferritin	AVEASLQLEK	3.177 (*p* = 0.0157)	8.417 (*p* = 0.0637)
		SLGDLLTNVR		
Unigene17945_All	minus strand odorant-binding protein RproOBP2 precursor	ELQVFGK	0.305 (*p* = 0.0254)	0.56 (*p* = 0.0555)
		VPENLGNPCEVAFAVTK ^a^		
		VPENLGNPCEVAFAVTK ^b^		

^a^ and ^b^ identified the same peptide sequence but with different valence states.

## Data Availability

Data is contained within the article or supplementary material.

## Supplementary Materials

The following supporting information can be downloaded at: https://www.mdpi.com/article/10.3390/insects13070605/s1, Text S1: PRM validation of differentially expressed proteins from iTRAQ; Figure S1: Pathway of insect hormone biosynthesis affected by improved artificial diet feeding in adults of *Arma chinensis*; Figure S2: Pathway of longevity regulating affected by improved artificial diet feeding in nymphs of *Arma chinensis*; Figure S3: Pathway of protein export affected by improved artificial diet feeding in nymphs of *Arma chinensis*; Figure S4: Pathway of biosynthesis of amino acids affected by improved artificial diet feeding in adults of *Arma chinensis*; Figure S5: Pathway of glycolysis/gluconeogenesis affected by improved artificial diet feeding in adults of *Arma chinensis*; Figure S6: Pathway of pentose phosphate affected by improved artificial diet feeding in adults of *Arma chinensis*; Figure S7: Pathway of pentose and glucuronate interconversions affected by improved artificial diet feeding in nymphs of *Arma chinensis*; Figure S8: Pathway of lysosome affected by improved artificial diet feeding in adults of *Arma chinensis*; Figure S9: Pathway of lysosome affected by improved artificial diet feeding in nymphs of *Arma chinensis*; Figure S10: Pathway of peroxisome affected by improved artificial diet feeding in nymphs of *Arma chinensis*; Table S1: MS/MS spetrum database search analysis summary; Table S2: GO functional classification of the up-regulated DEPs in IADA/COSMA; Table S3: GO functional classification of the down-regulated DEPs in IADA/COSMA; Table S4: GO functional classification of the up-regulated DEPs in IADN/COSMN; Table S5: GO functional classification of the down-regulated DEPs in IADN/COSMN; Table S6: GO enrichment for the up-regulated DEPs in IADA/COSMA; Table S7: GO enrichment for the down-regulated DEPs in IADA/COSMA; Table S8: GO enrichment for the up-regulated DEPs in IADN/COSMN; Table S9: GO enrichment for the down-regulated DEPs in IADN/COSMN; Table S10: KEGG pathway enrichment analysis for the up-regulated DEPs in IADA/COSMA; Table S11: KEGG pathway enrichment analysis for the down-regulated DEPs in IADA/COSMA; Table S12: KEGG pathway enrichment analysis for the up-regulated DEPs in IADN/COSMN; Table S13: KEGG pathway enrichment analysis for the down-regulated DEPs in IADN/COSMN; Table S14: Protein domain enrichment analysis for the up-regulated DEPs in IADA/COSMA; Table S15: Protein domain enrichment analysis for the down-regulated DEPs in IADA/COSMA; Table S16: Protein domain enrichment analysis for the up-regulated DEPs in IADN/COSMN; Table S17: Protein domain enrichment analysis for the down-regulated DEPs in IADN/COSMN; Table S18: DEPs associated with different biological characteristics; Table S19: DEPs associated with nutrients in improved artificial diet.
