# Supplementary material for: Differential Proteomics Analysis Unraveled Mechanisms of Arma chinensis Responding to Improved Artificial Diet
Source: Insects. 2022 Jul 2;13(7):605. doi: 10.3390/insects13070605 (PMC9319121; doi:10.3390/insects13070605)
Supplement: Supplementary file 1 [file insects-13-00605-s001.zip › Supplementary Figures.pdf]

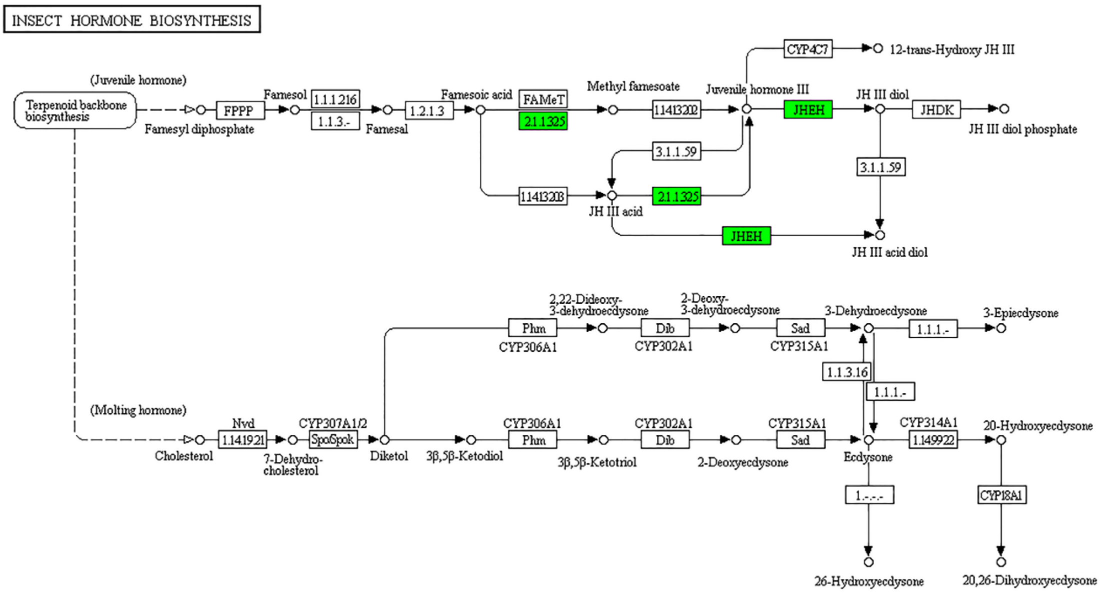

Supplementary Figure S1. Pathway of insect hormone biosynthesis affected by improved artificial diet feeding in adults of *Arma chinensis*. The red part indicates up-regulated proteins and the green part indicates down-regulated proteins.

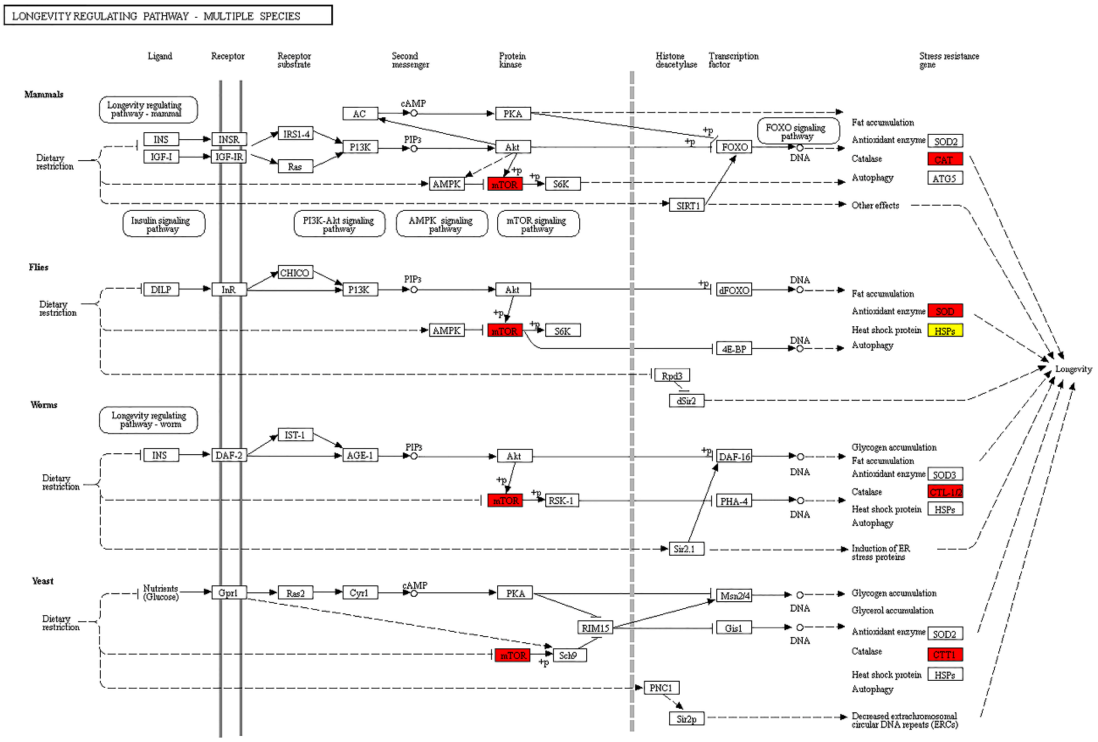

Supplementary Figure S2. Pathway of longevity regulating affected by improved artificial diet feeding in nymphs of *Arma chinensis*. The red part indicates up-regulated proteins and the yellow part indicates HSPs (Protein accession: Unigene17363_All, IADN/COSMN ratio = 2.707, p = 0.031; Protein accession: CL1793.Contig1_All, IADN/COSMN ratio = 0.621, p = 0.005).

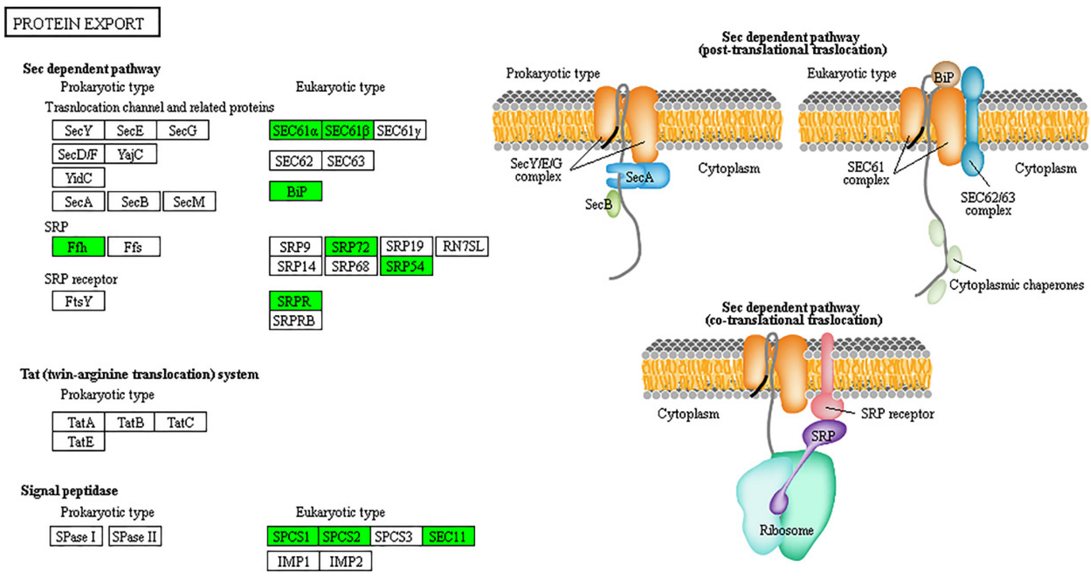

Supplementary Figure S3. Pathway of protein export affected by improved artificial diet feeding in nymphs of *Arma chinensis*. The green part indicates down-regulated proteins.

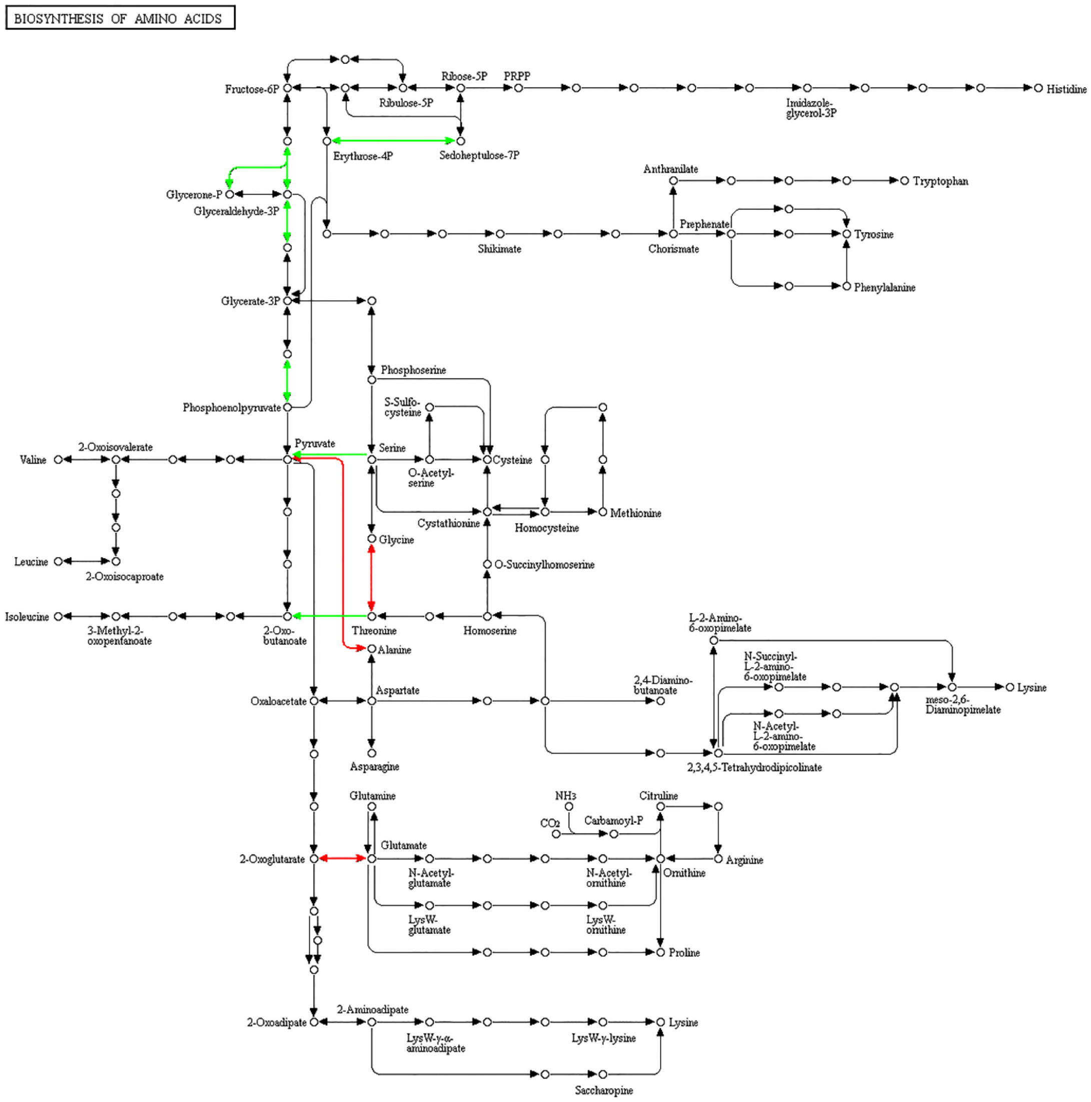

Supplementary Figure S4. Pathway of biosynthesis of amino acids affected by improved artificial diet feeding in adults of *Arma chinensis*. The red part indicates up-regulated proteins and the green part indicates down-regulated proteins.

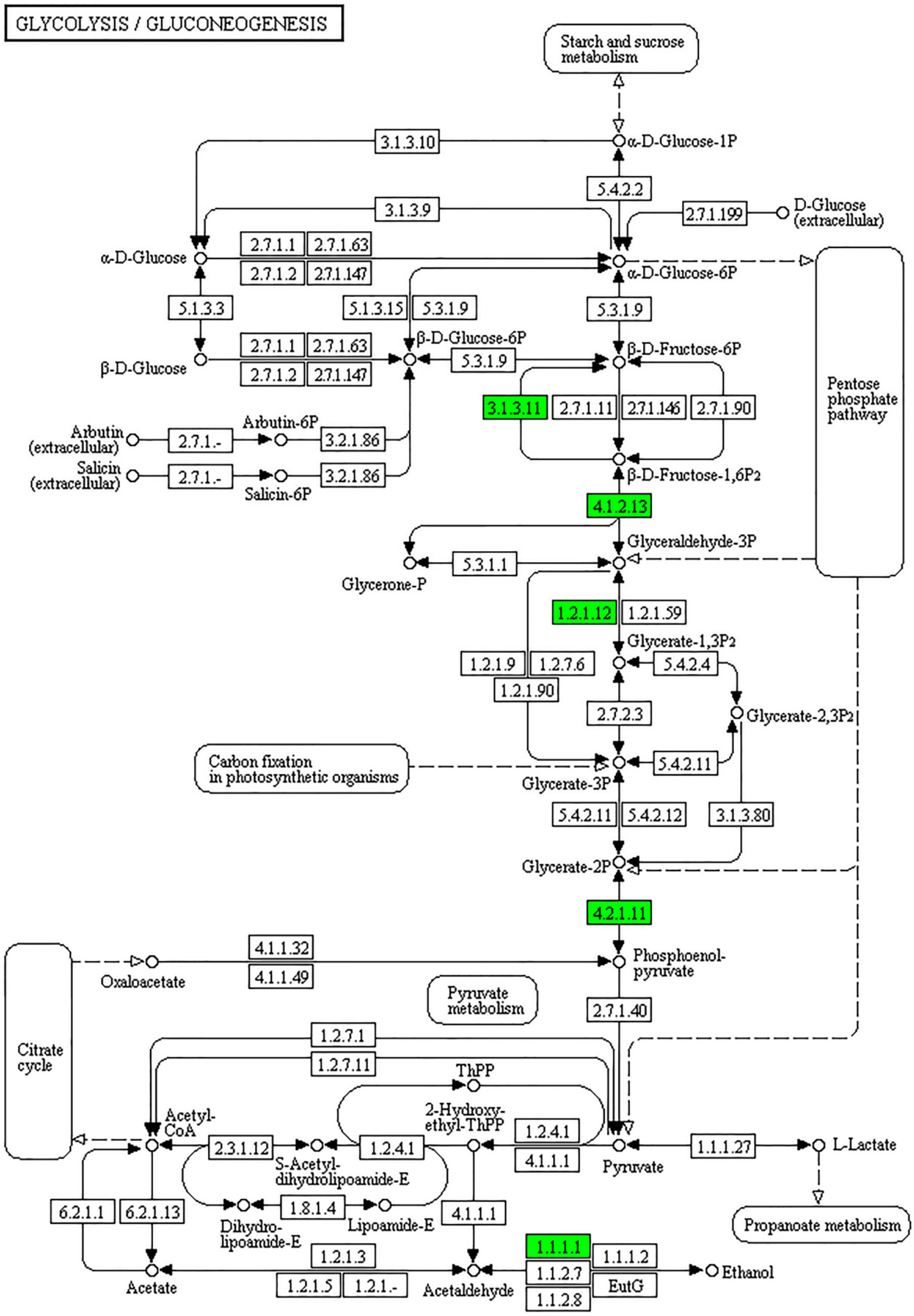

Supplementary Figure S5. Pathway of glycolysis/gluconeogenesis affected by improved artificial diet feeding in adults of *Arma chinensis*. The green part indicates down-regulated proteins.

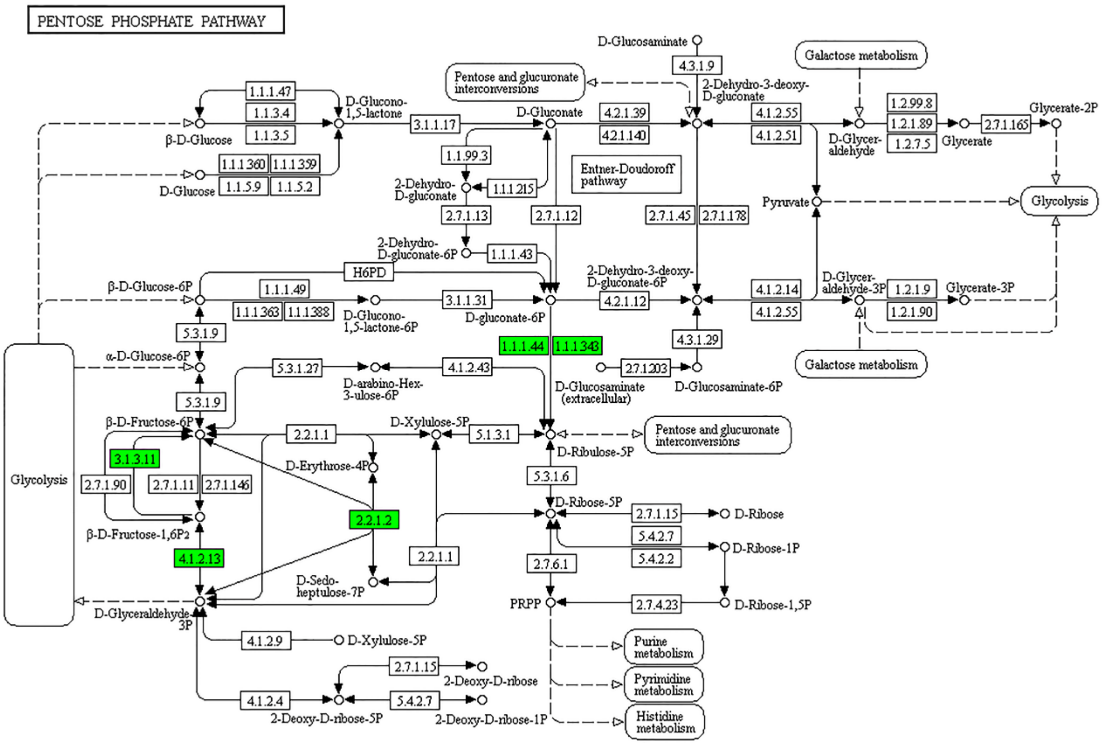

Supplementary Figure S6. Pathway of pentose phosphate affected by improved artificial diet feeding in adults of *Arma chinensis*. The green part indicates down-regulated proteins.

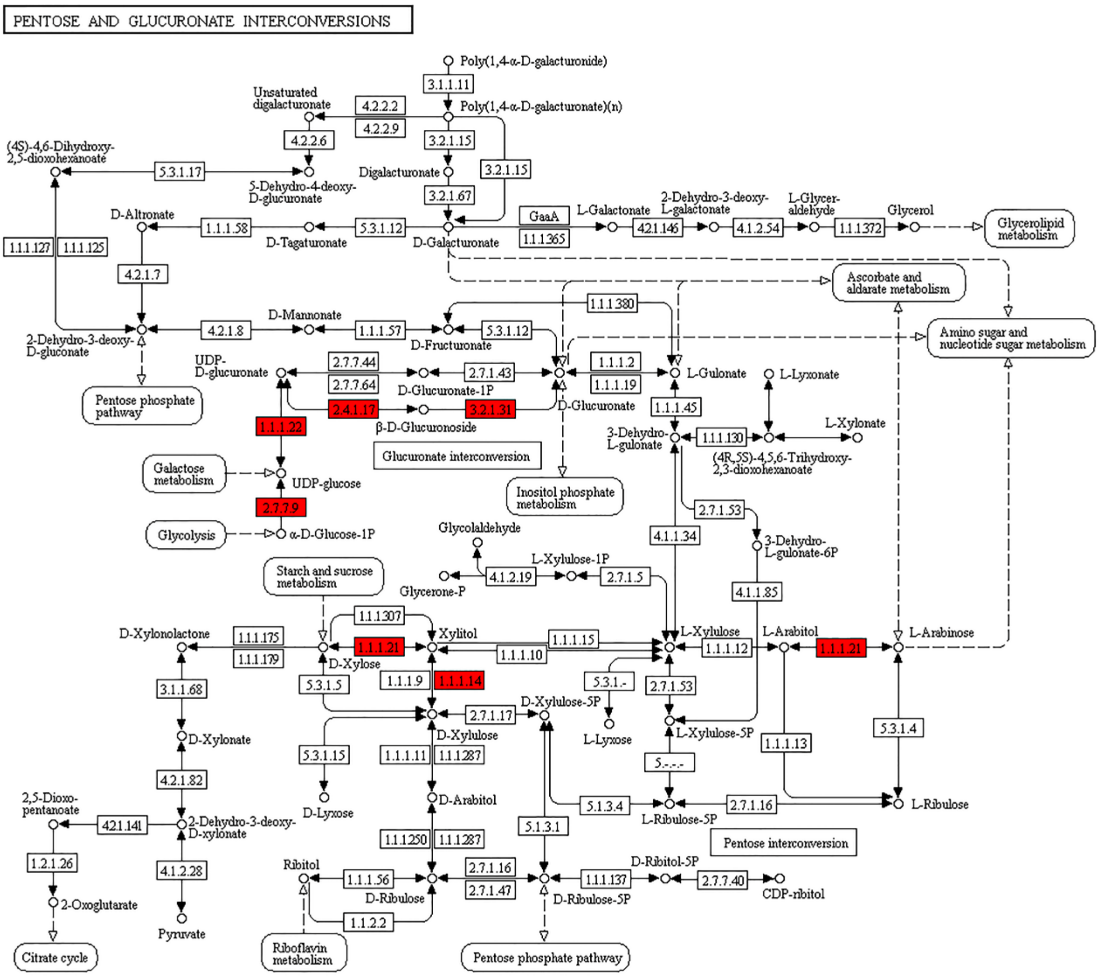

Supplementary Figure S7. Pathway of pentose and glucuronate interconversions affected by improved artificial diet feeding in nymphs of *Arma chinensis*. The red part indicates up-regulated proteins.

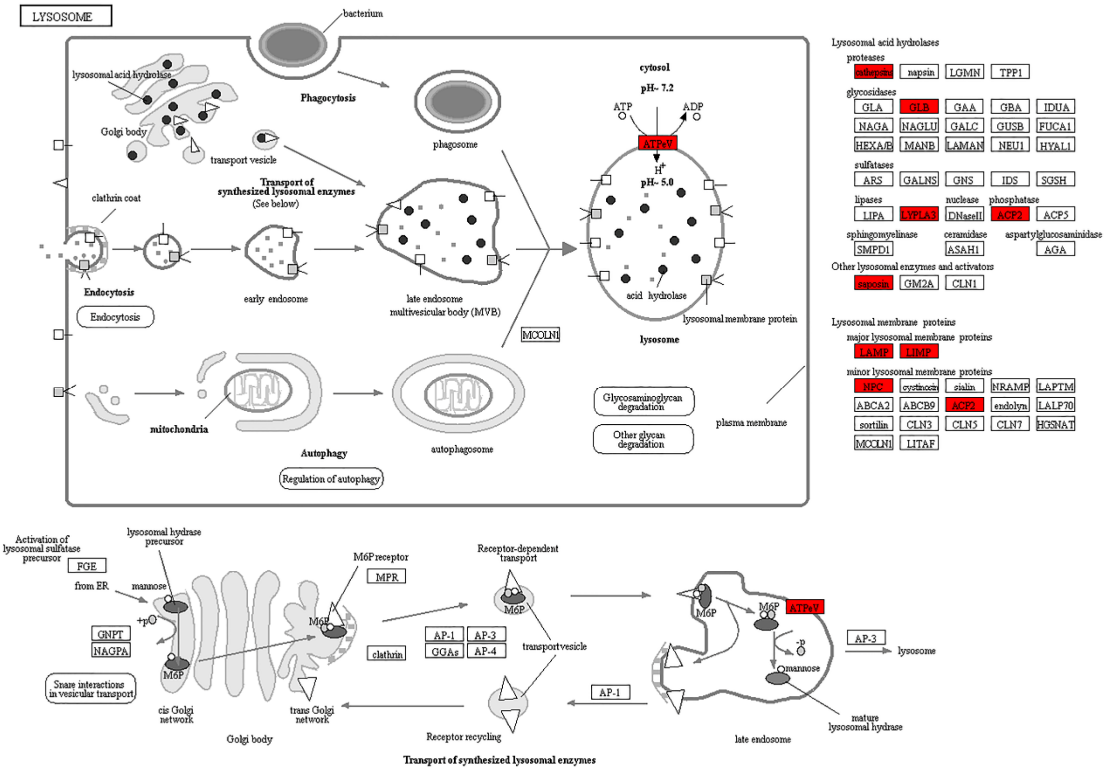

Supplementary Figure S8. Pathway of lysosome affected by improved artificial diet feeding in adults of *Arma chinensis*. The red part indicates up-regulated proteins.

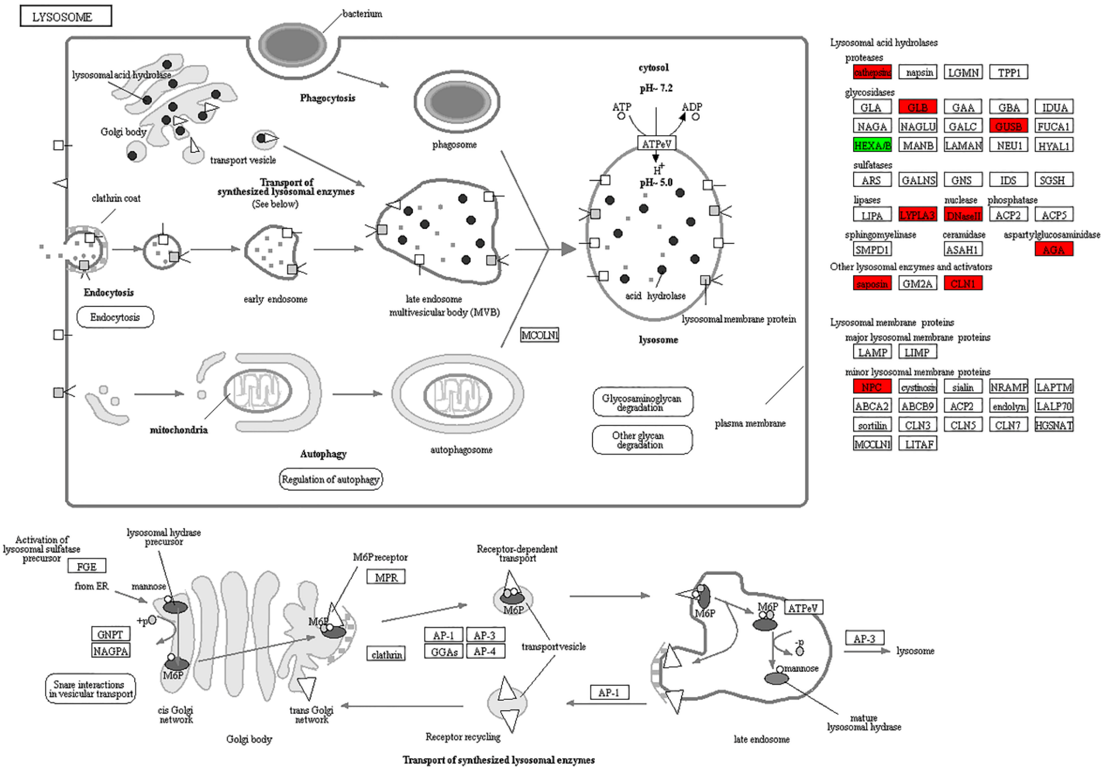

Supplementary Figure S9. Pathway of lysosome affected by improved artificial diet feeding in nymphs of *Arma chinensis*. The red part indicates up-regulated proteins and the green part indicates down-regulated proteins.

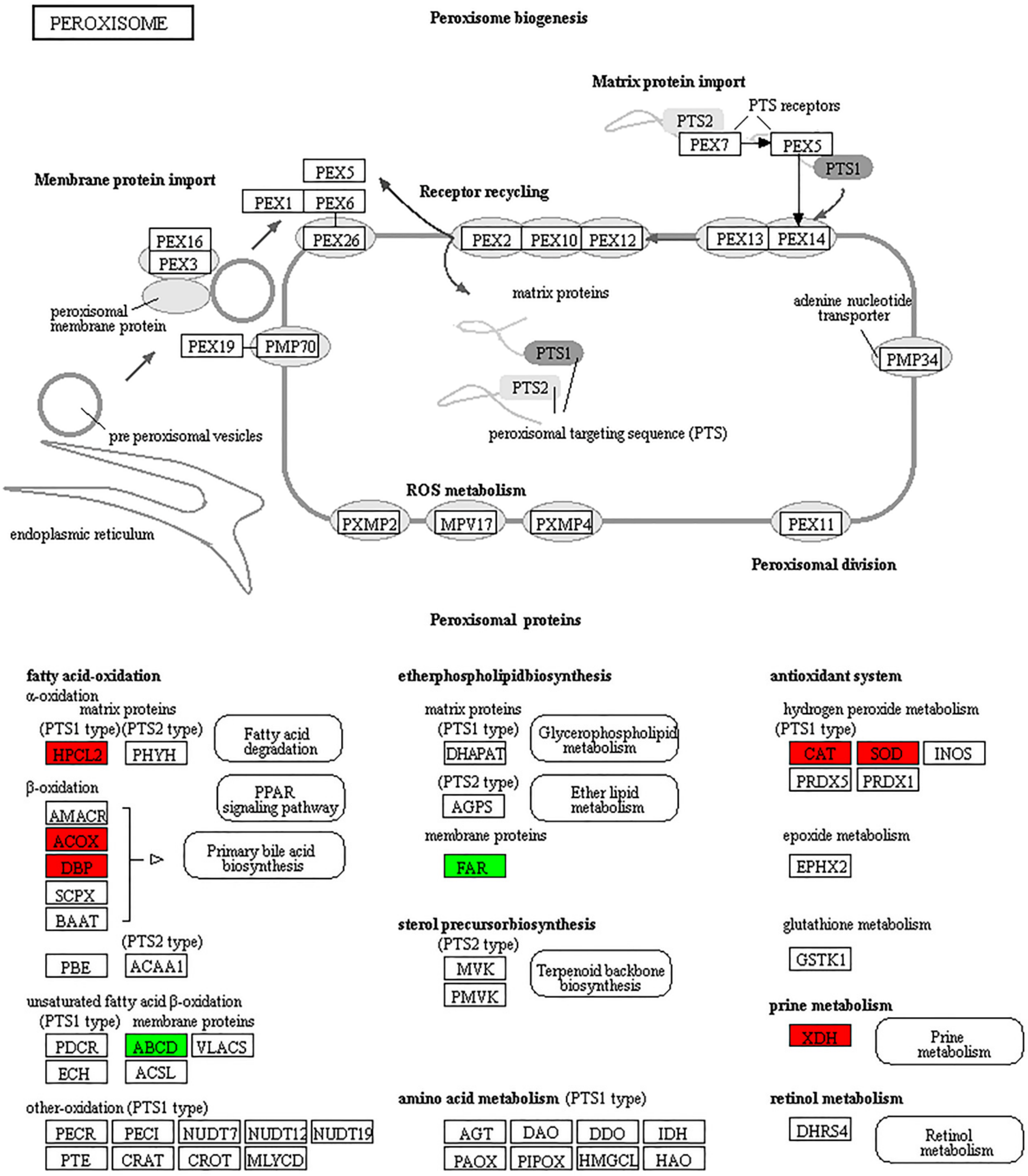

Supplementary Figure S10. Pathway of peroxisome affected by improved artificial diet feeding in nymphs of *Arma chinensis*. The red part indicates up-regulated proteins and the green part indicates down-regulated proteins.
