# Supplementary material for: Differential Proteomics Analysis Unraveled Mechanisms of Arma chinensis Responding to Improved Artificial Diet
Source: Insects. 2022 Jul 2;13(7):605. doi: 10.3390/insects13070605 (PMC9319121; doi:10.3390/insects13070605)
Supplement: Supplementary file 1 [file insects-13-00605-s001.zip › Supplementary Text S1.pdf]

Supplementary information

Differential Proteomics Unraveled Molecular Mechanisms of *Arma chinensis* Responding to Improved Artificial Diet

Deyu Zou [1], Thomas A. Coudron [2], Huihui Wu [3,*], Lisheng Zhang [4], Mengqing Wang [4], Weihong Xu [1], Jingyang Xu [1], Liuxiao Song, and Xuezhuang Xiao [3]

1 Mass Production Base of Natural Enemy Insects of Tianjin Academy of Agricultural Sciences, Institute of Plant Protection, Tianjin Academy of Agricultural Sciences, Tianjin 300384, China;
2 Biological Control of Insects Research Laboratory, USDA-Agricultural Research Service, Columbia, MO, 65203-3535, USA;
3 College of Horticulture and Landscape, Tianjin Agricultural University, Tianjin 300392, China;
4 Key Laboratory of Integrated Pest Management in Crops, Ministry of Agriculture, Institute of Plant Protection, Chinese Academy of Agricultural Sciences, Beijing 100193, China;

* Correspondence: Dr. Huihui Wu
No.22, Jinjing Road, Xiqing District, Tianjin 300384, China
E-mail: wuhuihui@tjau.edu.com Tel: 86-22-23781319

Supplementary Text 1.

PRM validation of differentially expressed proteins from iTRAQ

Protein Extraction

The sample was grinded by liquid nitrogen into cell powder and then transferred to a 5-mL centrifuge tube. After that, four volumes of lysis buffer (8 M urea, 1% Protease Inhibitor Cocktail) was added to the cell powder, followed by sonication three times on ice using a high intensity ultrasonic processor (Scientz). The remaining debris was removed by centrifugation at 12,000 g at 4 °C for 10 min. Finally, the supernatant was collected and the protein concentration was determined with BCA kit according to the manufacturer's instructions.

Trypsin Digestion

For digestion, the protein solution was reduced with 5 mM dithiothreitol for 30 min at 56 °C and alkylated with 11 mM iodoacetamide for 15 min at

room temperature in the dark. The protein sample was then diluted to urea concentration less than 2M. Finally, trypsin was added at 1:50 trypsin-to-protein mass ratio for the first digestion overnight and 1:100 trypsin-to-protein mass ratio for a second 4 h-digestion.

LC-MS/MS Analysis

The tryptic peptides were dissolved in 0.1% formic acid (solvent A), directly loaded onto a home-made reversed-phase analytical column (15-cm length, 75 μm i.d.). The gradient was comprised of an increase from 6% to 23% solvent B (0.1% formic acid in 98% acetonitrile) over 38 min, 23% to 35% in 14 min and climbing to 80% in 4 min then holding at 80% for the last 4 min, all at a constant flow rate of 400 nL/min on an EASY-nLC 1000 UPLC system.

The peptides were subjected to NSI source followed by tandem mass spectrometry (MS/MS) in Q Exactive™ Plus (Thermo) coupled online to the UPLC. The electrospray voltage applied was 2.0 kV. The m/z scan range was 350 to 1000 for full scan, and intact peptides were detected in the Orbitrap at a resolution of 35,000. Peptides were then selected for MS/MS using NCE setting as 27 and the fragments were detected in the Orbitrap at a resolution of 17,500. A data-independent procedure that alternated between one MS scan followed by 20 MS/MS scans. Automatic gain control (AGC) was set at 3E6 for full MS and 1E5 for MS/MS. The maxumum IT was set at 20 ms for full MS and auto for MS/MS. The isolation window for MS/MS was set at 2.0 m/z.

Data Analysis

The resulting MS data were processed using Skyline (v.3.6). Peptide settings: enzyme was set as Trypsin [KR/P], Max missed cleavage set as 2. The peptide length was set as 8-25, Variable modification was set as Carbamidomethyl on Cys and oxidation on Met, and max variable modifications was set as 3. Transition settings: precursor charges were set as 2, 3, ion charges were set as 1, 2, ion types were set as b, y, p. The product ions were set as from ion 3 to last ion, the ion match tolerance was set as 0.02 Da. Differences analysis in protein expression in the PRM-MS between the IAD and the COSM groups were performed using a T-test, and $P < 0.10$ was taken to indicate statistical significance.
